# Reply to Comments: Hurdle Clearance Detection and Spatiotemporal Analysis in 400 Meters Hurdles Races Using Shoe-Mounted Magnetic and Inertial Sensor

**DOI:** 10.3390/s20102993

**Published:** 2020-05-25

**Authors:** Mathieu Falbriard, Maurice Mohr, Kamiar Aminian

**Affiliations:** 1 Laboratory of Movement Analysis and Measurement, EPFL, 1015 Lausanne, Switzerland; kamiar.aminian@epfl.ch; 2 Institute of Sport Science, University of Innsbruck, 6020 Innsbruck, Austria; maurice.mohr@uibk.ac.at

## 1. Introduction

The current document answers the comment addressed by Schmidt, M., Nolte, K., Alt, T., and Jaitner, T. regarding our recent paper “Hurdle Clearance Detection and Spatiotemporal Analysis in 400 Meters Hurdles Races Using Shoe-Mounted Magnetic and Inertial Sensor” (Sensors 2020, 20, 354) [1].

First, the authors want to thank the aforementioned researchers for their comments and constructive feedback. We acknowledge the importance of ecological validity and therefore appreciate any response aiming to improve the proposed system. Moreover, we emphasize that the comments are concerned with our secondary study aim, i.e., monitoring contact time (CT) and flight time (FLY) throughout the race. Our primary aim was to detect hurdle crossing events, identify the leading leg, and estimate the average speed between the hurdles, which was accomplished with high accuracy.

Although we agree with most of the limitations discussed in the comment by Schmidt, M., Nolte, K., Alt, T., and Jaitner, T., we think that it is necessary to clarify some of the key arguments. Therefore, we have divided our answer into three parts: (1) underestimation of CTs, (2) adaptations applied to the algorithms presented in [2], and (3) the need for in-field validation.

## 2. Response

### 2.1. Underestimation of CTs

As we discussed in [2] and was appropriately reported in the comments, the Inertial Measurement Unit (IMU) based system underestimates CT, with a bias that depends on the running speed. Although we were aware of these results in [1], we did not correct the bias for CT estimations for the following reasons:

- The range of speeds observed in [1] was greater than for the validation study [2]; therefore, we could not obtain the actual bias at the observed running speeds.
- The results from [2] indicate that the bias decreases as the running speed increases. Hence, we extrapolated the linear trend of the bias and assumed that it would be relatively low at the running speeds observed in [1].
- The correction based on the running speed would require a measure of the instantaneous speed to apply the appropriate correction at each step. The current study [1] estimates the average running speed between the hurdles. We expected the speed to vary between the hurdles and therefore did not use the average speed to reduce the bias of CT and FLY.

Nevertheless, we agree with the comments and acknowledge the observation that the reported CTs are relatively low. We should have discussed this lack of correction in [1] and thank the authors of the comments for their remark. We will investigate the different options available to estimate the bias based on other metrics. However, we want to point out that the speed-dependent biases should not have a significant effect on the trends observed in Figure 8 of [1] since the trend is consistent among the different subjects.

### 2.2. Adaptations Applied to the Algorithms 

**Figure 1:**
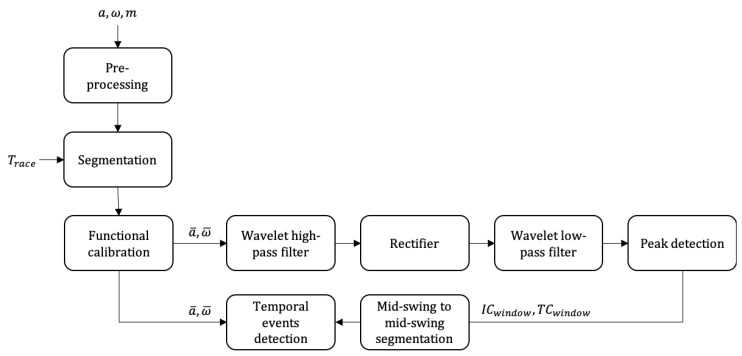
The processing steps added before temporal events detection to improve the segmentation in mid-swing to mid-swing periods.

As we mentioned in [1], the detection algorithm was affected by the noise due to the hurdle clearance movement, the adaption steps before and after the hurdle, and also by the high running speeds. However, the noise affected the robustness of the mid-swing events detection algorithm (used to segment the signal in mid-swing to mid-swing cycles), not the detection of initial contact (IC) and terminal contact (TC) (Figure 1). Thus, the wavelet filter procedure in [1] was simply applied to define a time window in which IC and TC had to occur. Then, we detected initial and terminal contact within this window using the method validated in [2] (i.e., the detection of the temporal events was not based on the output of the wavelet filters). Consequently, these adaptations should not affect the estimation of CT and FLY. 

### 2.3. Need for In-Field Validation

We concede that our system was validated for treadmill running and could further be improved with validation in real-world settings. However, the kinematics feature used to detect mid-swing events as well as initial and terminal contact events depend on the shape of the signal rather than on fixed thresholds. In [1], IC and TC were defined as the local minimum of the angular velocity in the sagittal plane before and after a mid-swing event. Since the rotation pattern of the foot in the sagittal plane is similar during overground running, we assume that these two minima can be generalized to outdoor running. Nevertheless, the bias of the system might change slightly between treadmill and overground running.

## 3. Conclusions

In conclusion, we agree with the comments from Schmidt, M., Nolte, K., Alt, T., and Jaitner, T. regarding the CTs reported in our study [1]; the CTs observed are likely to underestimate the exact duration of the stance phase. Based on the results from the validation study, a speed-specific correction of the bias should be applied. However, in our field study, the instantaneous speed was not available, and the bias could not be corrected accordingly. Apart from this observation, we argue that the proposed system can be used to provide an analysis of 400 meters hurdling in real-world conditions and that systematic error (bias) should not affect the trends presented in Figure 8 of [1]. Also, the proposed methods for hurdle crossing detection, leading leg identification, and average speed between the hurdles remain valid.

## References

[1] Falbriard M., Mohr M., Aminian K. (2020). Hurdle Clearance Detection and Spatiotemporal Analysis in 400 Meters Hurdles Races Using Shoe-Mounted Magnetic and Inertial Sensors. Sensors.

[2] Falbriard M., Meyer F., Mariani B., Millet G.P., Aminian K. (2018). Accurate estimation of running temporal parameters using foot-worn inertial sensors. Front. Physiol..

